# Telemedicine in Tinnitus: Feasibility, Advantages, Limitations, and Perspectives

**DOI:** 10.1155/2013/218265

**Published:** 2013-05-14

**Authors:** Matthieu J. Guitton

**Affiliations:** ^1^Department of Otorhinolaryngology and Ophthalmology, Faculty of Medicine, Laval University, Quebec City, QC, Canada G1V 0A6; ^2^Institut Universitaire en Santé Mentale de Québec (IUSMQ), 2601 chemin de la Canardière F-6517, Quebec City, QC, Canada G1J 2G3

## Abstract

Given the important patient needs for support and treatment, telemedicine—defined by medical approaches supported by the new technologies of information—could provide interesting alternative in tinnitus treatment. By analyzing the published tools and approaches which could be used in the context of telemedicine for tinnitus by health professionals or self-administrated by patients, this review summarizes, presents, and describes the principal telemedicine approaches available presently or in the near future to help assess or treat tinnitus or to offer support to tinnitus sufferers. Several pieces of evidence strongly support the feasibility of telemedicine approaches for tinnitus. Telemedicine can be used to help tinnitus sufferers at several points in the therapeutic process: for early screening, initial evaluation, and diagnosis; for optimizing therapeutic tools, particularly behavioural therapies and virtual reality-enhanced behavioral therapies; for long-term monitoring of patients and provision of online support. Several limitations are, however, discussed in order to optimize the safe development of such approaches. Cost effective and easy to implement, telemedicine is likely to represent an important part of the future of tinnitus therapies and should be progressively integrated by otolaryngologists.

## 1. Introduction

With the aging of the population and the increase in noise-pollution levels, tinnitus—the perception of sound in silence—is affecting a growing number of people. This condition represents a heavy burden for the sufferers and their relatives and a major problem for public health. Despite important advances in the last decade in the development of pharmacological strategies using animal models to block the perception of tinnitus [1–5], these results have not yet been fully translated into easily available clinical solutions [6]. Psychological and behavioral support unfortunately remains the main therapeutic strategy for tinnitus. However, in the context of limited resources, otolaryngologists, audiologists, or psychologists simply cannot provide tinnitus sufferers with the time and attention they would need to cope with their symptoms. Thus, once a diagnosis has been made and dramatic aetiologies have been ruled out (e.g., neurinoma), the health specialist is often left with no other solution than to tell to the patient to learn to “live with his tinnitus.”

Therefore, in the absence of effective pharmacological treatment, the vast majority of tinnitus sufferers are left to themselves. Hence, tinnitus must often be managed as a chronic problem, with therapeutic strategies still relying heavily on time-consuming behavioral approaches [6, 7].

Recent advances in information technologies provide new opportunities to enhance the patient-clinician relationship through assistance at a distance. “Telemedicine” allows to save costs, to save time, and to save resources, and it is particularly well adapted for situations in which medical resources are limited, as well as when patients need long-term support. As a pathology, tinnitus perfectly exemplifies the main and classical advantages of telemedicine.

Here, we will summarize the possibilities offered by telemedicine in the context of management of tinnitus sufferers, from initial patient screening and diagnosis to online group support and possible teletreatments. We will then discuss the advantages of telemedicine approaches in the context of tinnitus, evaluate the feasibility of such approaches, explore some of their limitations, and finally mention some of the perspectives on telemedicine strategies for tinnitus.

## 2. Online Assessment

Although it is not yet common in medical practice, telemedicine, whether for distance diagnosis or distance intervention, is becoming increasingly important. The diagnostic use of telemedicine is particularly interesting. Indeed, this approach provides numerous advantages: it offers the expertise of specialists to remote population, it reduces the costs and waiting times of preclinical or clinical evaluation, and it allows efficient screening to be performed. Some medical specialties, such as cardiology, rely heavily on use of diagnostic telemedicine [8], but numerous other applications exist. For instance, skin disorders occurring at sea and requiring acute treatment can be accurately diagnosed and effectively treated using telemedicine services [9].

**Figure 1:**
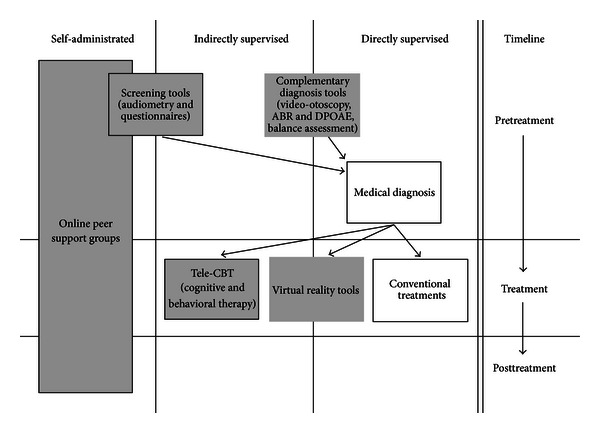
Graphic chart of the sequential use of telemedicine for tinnitus. This flowchart demonstrates the different steps of tinnitus management on a clinical timeline, according to the way the applications are administrated: self-administrated by patients without medical supervision, indirectly supervised by remote health care professionals, or directly supervised in a “face-to-face” conventional setting. This figure displays the hierarchical use of telemedicine tools for tinnitus. Empty boxes represent conventional interventions. Grey-filled boxes represent telemedicine interventions already available. Grey-framed boxes represent telemedicine interventions not yet easily available.

**Figure 2:**
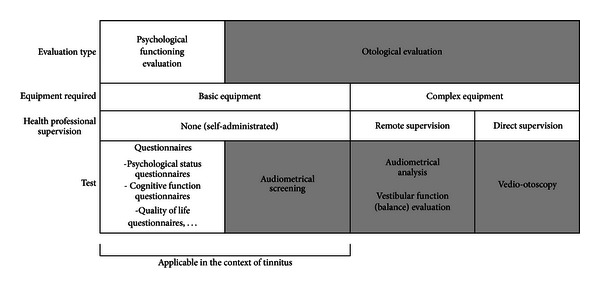
Telemedicine in tinnitus diagnosis. This figure demonstrates the different tools which can be used in the context of telediagnosis of tinnitus. Two main categories of evaluations can be performed: psychosocial functioning evaluations (white) and specific otological evaluations (grey). Basic equipment refers to a computer, Internet access, and a set of earphones, while complex equipment refers to a supplementary specific apparatus, such as a high resolution camera for balance assessment. Direct supervision by a health professional refers to the actual presence of this professional with the patient (e.g., in the case of video-otoscopy, for which a professional needs to position the camera inside the ear of the patients to collect the pictures which will be analysed remotely later on). The applications which can already be used for tinnitus assessment are mentioned under the “Applicable in the context of Tinnitus” label.

Despite its potential for diagnosis, telemedicine is unfortunately surprisingly underdeveloped in the field of otolaryngology. However, several elements of the process of tinnitus diagnosis and evaluation are particularly well suited for telemedicine applications. Before any *bona fide* medical intervention is considered, the first application of telemedicine in the therapeutic process is for diagnosis (Figure 1). Distance investigation of the auditory system, such as video-otoscopy, objective measures of auditory functioning with ABR or DPOAE recordings, or balance assessments are possible and show good reliability compared to face-to-face examination [10–15]. However, such procedures require equipment not likely to be found in a patient's home and often also require the presence of a specialist, either with the patient (for video-otoscopy) or in a remote location to monitor the patient (for balance assessment). Thus, these procedures may be more suited to remote interventions rather than in the context of general tinnitus patient care (Figure 2).

Telemedicine can nonetheless find its use in screening tinnitus patients. Although complete audiometric analysis requires the expertise of a specialist, audiometric screening can be self-performed by patients using very simple equipment, that is, a computer, a set of headphones, and appropriate simple software [15]. Proof-of-concept of such a strategy has already been reported by a few groups. Self-test Internet-based audiometry can be done for pure tone, such as frequencies between 500 and 8,000 Hz [16], or with more complex stimuli, such as speech-in-noise screening [17, 18]. Although legitimate questions regarding the acoustical control in such procedures can arise (transducer type and calibration, noise levels in the patients home, etc.), these tools still show very good reliability for hearing screening tests and are both time and cost effective for the health care system [18].

One of the problems in dealing with tinnitus sufferers is the commonly observed dissociation between the objective medical reality and the psychological, perceived reality of the patient. In many cases, tinnitus with no known aetiology other than the history of noise trauma or general aging of the auditory system—which would not be considered as a sign of more dangerous pathologies and would not represent a medical priority *per se*—is often perceived as extremely invasive and distressing by the patients, who expect medical doctors to provide them with support in one form or another [19]. However, without proper tools to objectively assess such a phenomenon, it may be difficult for otolaryngologists to quickly identify these patients who should be examined to avoid dramatic outcomes such as suicide. Internet-delivered quality of life questionnaires could represent extremely valuable tools to help identify the most vulnerable patients—those who would need to be assessed and helped in priority, in addition to those for whom tinnitus is a sign of a more problematic medical issue.

Questionnaires—either aimed at assessing general psychological or social status, cognitive functions, or quality of life—can provide extremely valuable information. It has been demonstrated that the results obtained from online-delivered questionnaires are comparable to those obtained in classical pen and paper settings [20, 21]. Furthermore, anxiety and depression questionnaires have been found to provide reliable information when delivered online to tinnitus sufferers [22]. Hence, online self-administrated questionnaires can provide valid data which can help to identify accompanying symptoms and assist in orienting the diagnosis to a particular aetiology (Figure 1).

When considering online assessment and teleevaluation, it is, however, important to consider and always keep in mind that distance evaluation cannot replace a complete “face-to-face” medical examination (and is not aimed at doing so either), but rather enables to sort the patients by order of priority and to eventually orient and direct the patients to the required complementary examination more quickly. Once the otolaryngologist finally meets the patient face-to-face, he already has in his hands not only the general screening results obtained through telemedicine diagnosis platforms, but also the results of the complementary exams requested following the initial distance screening procedures.

## 3. Online Peer Support Groups

One of the main problems of chronic health conditions such as tinnitus is the associated loneliness accompanying the debilitating symptoms [23]. New information technologies and networks supported by the Internet provide novel opportunities for patients who share similar health conditions to access peer support. Online support groups—forums, blogs, and other dedicated sites—are popular web-based resources which can offer both tailored information and peer support through virtual communities. Online support groups have been shown to be beneficial for patients suffering from a wide range of chronic health conditions, including but not limited to cancer, diabetes, chronic respiratory diseases, arthritis, or weight control problems [24–28].

Despite often presenting high dropout rates [27], online support groups have been shown to increase confidence and self-management abilities for some patients suffering from chronic diseases [24–26]. In addition, such online support groups have been demonstrated to significantly decrease patients' loneliness, resulting in a clear improvement in subjective well-being and quality of life [28]. Obviously, all these effects are extremely interesting in the particular context of tinnitus. Interestingly, the benefits of participation seem to be rather similar between people participating in online peer-support groups and patients engaging in face-to-face support groups [29, 30].

In the case of tinnitus, for which the symptoms cannot be directly experienced by others than the sufferers, online support groups provide an extremely interesting way to break the social isolation triggered by tinnitus. However, it has a cost as well. Indeed, despite the aforementioned advantages, online support groups for tinnitus raise a few questions and issues, when considering their therapeutic potentials. The first major issue with online peer support groups is the lack of control over the quality and reliability of the information provided. Erroneous information could easily get propagated in a community of sufferers, eager for a cure [31]. This could be particularly damaging in the context of tinnitus, for which no pharmacological treatment effective in 100% of the cases is available, and for which “pseudotherapies” flourish. A second issue is that such online peer support groups may increase for the patients the feeling of being “abandoned” by the regular medical community. Finally, a third issue, particularly problematic in the context of tinnitus, is the risk that constantly focusing and discussing one's own tinnitus may have a “vicious effect” by enhancing the reinforcement of tinnitus by anxiety [6].

With the aim of developing telemedicine applications for tinnitus, it is extremely important not to underestimate the value of online peer support groups. Being low cost and easy to access from any computer with an Internet connection, online support groups can reach a broad population. Furthermore, they overlap with all points of the therapeutic process, as patients can and are likely to consult them—and to interact with the support group community—before, during, and after their treatment (Figure 1). Hence, despite their positive outcomes, there is still a need to critically evaluate and to optimize the online resources for tinnitus sufferers.

## 4. Online Therapies for Tinnitus

Although online peer support groups can provide valuable psychological help, they are clearly not enough to alleviate the debilitating symptoms. Therapeutic actions need to be taken in order to provide relief for sufferers. As mentioned in Section 1, this is a problematic issue concerning tinnitus, for which pharmacological treatments are still lacking. However, behavioral interventions based on cognitive behavioral therapy (CBT) have been repeatedly found to have positive effects on the capacity of sufferers to cope with their symptoms [32–35]. Unfortunately, there is a shortage of trained health professionals able to carry out such therapy. With the budget restrictions affecting public health systems in most Western countries, this situation will probably not get better in the near future.

Here again, telemedicine can provide valuable approaches. Although most studies have focused on face-to-face interventions, CBT is well suited for distance interventions as well. CBT for tinnitus sufferers can be performed either using self-help books based on CBT (sometimes referred as “bibliotherapy”) [36, 37], or Internet-based interactive material supporting self-administrated CBT [38]. Positive effects to reduce tinnitus sufferers' distress have been demonstrated for such approaches [36–39]. An alternative solution is to combine self-administrated intervention with distance guidance in the form of brief telephone support from health professionals [40]. Interestingly, effectiveness of self-help treatments is relatively comparable to face-to-face group CBT and is almost up to five times as cost effective as conventional CBT treatment [40]. Furthermore, Internet-delivered CBT seems to have long-lasting effects, as assessed by 1-year patient follow-up [40]. An interesting randomized controlled trial investigating the effects of CBT and acceptance and commitment therapy (ACT) in an Internet-delivered guided self-help format demonstrated that Internet-delivered acceptance-based procedures could also be a viable alternative to regular CBT approaches in the case of tinnitus sufferers [38].

A common problem faced by Internet-delivered therapy is adherence to treatment. Tinnitus sufferers, however, present an interesting case in this regard, since they are eager to get relief from their symptoms and, therefore, more likely to stay in a program with documented positive outcomes. In accordance with this assumption, patients' views and acceptance of such Internet-delivered programs are positive [39]. A way to enhance adherence to Internet-based cognitive intervention is to integrate it with an online support group [41]. This strategy, tested for cancer survivors, has a very high degree of feasibility for tinnitus, given the willingness of tinnitus patients to participate in support groups.

## 5. Virtual Reality-Enhanced Behavioral Therapies

In many cases, the main challenge for tinnitus therapists is to “retrain” the patients to operate normally—or at least, optimally—in a social context. The use of virtual environments is a promising avenue in the context of rehabilitation of tinnitus patients. Indeed, three-dimensional (3D) computerized virtual settings are inherently multimodal [42–44] and thus offer new possibilities for behavior-based therapies [45, 46]. In a virtual space, patients can be exposed to ecologically valid settings in a safe, controlled and convenient environment, such as a clinic [45, 46]. With integrated representations of multiple sensory dimensions of real-life experience, virtual settings have been successfully considered as possible complementary therapeutic tool for the treatment of pathologies including perceptual distortions, such as anorexia—which encompasses both taste-related perceptual distortions and body image distortions [46]. Based on pathological sensory processing, tinnitus could be a promising potential target for therapeutic strategies based on the use of virtual tools.

The social dimension of virtual spaces [47, 48] can also have interesting therapeutic implications [46]. The perception of tinnitus leads to significant disturbances of social behaviour [19, 23, 49], which have been replicated in animal models [50]. In virtual settings, patients can confront distressing social situations and manage their emotional and behavioral responses under the control of a therapist, without having to face actual deleterious consequences or the related social stress. Thus, in addition to other conventional therapies, such approaches involving virtual settings—face-to-face or in a distance telemedicine context—may offer an interesting option in the case of tinnitus.

## 6. Conclusions

Several pieces of evidence strongly support the feasibility of telemedicine approaches for tinnitus. Telemedicine for tinnitus can be implemented at different stages: diagnosis and initial evaluation, long-term follow-up and online support, behavioral therapies, and virtual reality-supported behavioral therapies.

Although in an ideal setting all the different telemedicine approaches could be integrated together, practically, this implementation could be more realistically done step by step. Indeed, in the context of tinnitus, each of these steps (screening, diagnosis, treatment, and long-term follow-up) is independent and often requires different health care professionals (audiologists, otolaryngologists, clinical psychologists, etc.). Furthermore, most telemedicine applications for tinnitus only require a computer, access to the Internet, and a set of earphones. A few steps can be identified to advance the applicability of telemedicine for tinnitus (Table 1).

Despite its clear and unquestionable potential, several elements, however, limit the actual application of telemedicine for tinnitus. Online audiometric screening could be self-administrated, saving time and costs for health system, but a full audiometric analysis would still require an audiologist. Similarly, even if some of the clinical exams can be done in remote settings, the diagnosis itself still has to be done by an otolaryngologist. Despite being often linked to noise-induced or age-related hearing loss, tinnitus can also be a symptom of numerous disorders, some of them potentially highly problematic, which could be ruled out only by a professional. Similarly, even though it is *a priori* easy to fill out a questionnaire, some assistance may be required if one of the questions is misunderstood.

Alternative solutions need to be found to respond to the growing demand from patients. Although telemedicine approaches will not address all the issues, they will for sure contribute to solving the problem of the saturation of the health care system. From this perspective, the use of Internet-delivered treatments and of virtual reality-enhanced settings is likely to represent the future of tinnitus-oriented behavioral therapies. Telemedicine in tinnitus, with both high feasibility and the possibility of implementing the different therapeutic steps individually, could serve as a testing ground for the more general use of telemedicine in otorhinolaryngology at large.

## Figures and Tables

**Table 1:** Next steps to face the challenges of telemedicine for tinnitus.

Type of intervention	Challenges
Teleaudiometry	Development and validation of software allowing calibrated and reliable teleaudiometry
Online support groups	Development of support material for patients
Basic behavioral therapy tools	Development of innovative behavioral therapy settings, self-explanatory, easy to use, and attractive and entertaining for patients (to increase adherence and avoid dropout)
Advanced behavioral therapy tools	Integration of behavioral therapy concepts within virtual settings
